# Floatable Syntactic Magnesium Foam as a Marangoni-Induced Propulsion Microboat

**DOI:** 10.3390/ma18245588

**Published:** 2025-12-12

**Authors:** Gyorgy Thalmaier, Niculina Argentina Sechel, Ioan Vida-Simiti

**Affiliations:** 1 Material Science and Engineering Departament, Materials and Environmental Engineering Faculty, Technical University of Cluj-Napoca, 103 Muncii Blv., 400641 Cluj-Napoca, Romania; niculina.sechel@stm.utcluj.ro (N.A.S.); vida.simiti@stm.utcluj.ro (I.V.-S.); 2 Institute of Nanomaterials and Nanotechnologies—Eutinn, European University of Technology, European Union, 28 Memorandumului Street, 400114 Cluj-Napoca, Romania; 3 Technical Science Academy of Romania, Dacia Ave., 26, 010413 Bucharest, Romania

**Keywords:** Marangoni propulsion, syntactic foam, magnesium foam, micro-swimmer

## Abstract

This study reports the successful fabrication and application of floatable syntactic foams derived from fine magnesium powder (<45 µm) utilizing expanded perlite (0.25 g/cm^3^, 0.2–0.4 mm) as the pore former. Sample disks with densities as low as 0.9 g/cm^3^ were produced via the classical press and sinter process. To ensure reasonable mechanical properties, the specimens were formed under a pressure of 200 MPa in a hardened steel die, followed by high-vacuum sintering (~3 × 10^−6^ torr) at 640 °C for 1 h. The resulting foams exhibited sufficient mechanical strength to allow for precision machining into a microboat. We demonstrated their potential use as a Marangoni-induced microswimmer. Spontaneous locomotion was observed when ethanol was used as a propellant, which generates a surface tension gradient between the upper and rear parts of the swimmer. The microboats achieved propulsion speeds of approximately 160 mm/s when propelled by a 95% ethanol + 5% ink mixture. Using a small volume (~4 µL) of the alcohol mixture, the swimmer could cover distances exceeding 350 mm.

## 1. Introduction

Metallic or ceramic foams are advanced cellular materials with a specific alveolar structure. From a physical point of view, these types of materials can be considered biphasic: one solid phase and one porous phase. The solid phase represents the skeleton of the foam structure, which can be made of metallic or ceramic materials. It ensures the shape of the part, the thin walls of the pores and the load-bearing capacity for various external stresses [1,2,3]. The porous phase consists of the network of closed pores (closed-cell foams) and open, intercommunicating pores (open-cell-intercommunicating foams) that surround the solid matrix.

The main structural characteristics of metal foams are their low density and very high porosity, generally over 70%, which give them the characteristic of being very light materials with a large specific surface area. This specific structure determines their special functional properties, such as their high capacity to absorb mechanical energy under impact loads or acoustic insulation. Thermal conductivity is low [4,5]. Such specific functional characteristics allow them to have various applications, such as orthopedic implants in medicine, parts subjected to mechanical impact shocks in automobile construction, heat exchangers and accumulators, floating elements, etc. [6,7].

The main materials used in the manufacture of metal foams are usually low-density metals and their alloys: aluminum, magnesium, tantalum, titanium, or alloys with specific properties, like nickel, zinc, and some stainless alloys.

In the specialized literature related to metallic and ceramic foams, a great number of books, monographs, and review articles have been published that contain definitions of the porous structures specific to foams, descriptions, and classifications of foam categories and of the methods and technological processes for obtaining them, of their specific properties, characteristics, and comparative functional performances, and of the applications of these types of advanced materials [8,9].

Powder metallurgy processes offer the possibility of obtaining small and medium-sized foam parts of complex shapes through press-free forming, classic press forming, sintering, hot isostatic pressing of powder mixtures with a pore-forming agent, and 3D printing.

R. Thiyagarajan, in the paper “A Review on Closed Cell Metal Matrix Syntactic Foams: A Green Initiative towards Eco- Sustainability” [10], presents a comprehensive synthesis on metal matrix syntactic foams. These foams are a special group of closed cell foams where the porosity is formed using hollow particles or other, mainly ceramic, high-porosity particles. The main metals used as the matrix are Al, Mg, Ti, Zn, Fe, and Cu. The main technological approaches for the manufacture of syntactic foams are presented comparatively, highlighting the powder metallurgy route with application potential, along with the influence of the technological process on some functional properties that can ensure applications in the aeronautical, automotive, construction, and defense industries.

It is worth noting that metallic foams can be obtained from waste materials, and interesting experimental results were obtained using recycling aluminum chips [11,12].

Syntactic foams with a Mg matrix have a high potential for application in the construction of aircraft and automobiles [13].

Intensive research is being focused on obtaining composite and hybrid syntactic foams by applying cheap pore formers of mineral rocks such as perlite (Al_2_O_3_-SiO_2_-based ceramic). In previous works [14,15], the experimental results on the effect of chemical compounds formed during the processing of syntactic Mg foams with perlite particles, respectively, of Mg and Al foams with (Al_2_O_3_/SiO_2_/mullite) particles, on the behavior under compression loads are presented. A hardening process of the foams with an effect on the specific compression characteristics is observed. The influence of the manufacturing conditions (space holder content—namely, carbamide—and sintering regime) of magnesium foam on microstructural properties resulting from the sintering dissolution process (SDP) is presented in the work of S.F. Aida et al. [16]. Mg foam with 50 wt carbamide % obtained at a sintering temperature of 630 °C has optimum properties compared to other compositions of carbamide for all sintering temperatures.

A large, in-depth and comparative study on advanced research on the fabrication processes, mechanical properties, and corrosion behavior of magnesium matrix syntactic foams is presented in [17]. The syntactic Mg foams have low densities compared to the metallic and polymeric foams with interconnected pores. The experimental results show their superior mechanical and energy absorption properties, which give them, among other things, possible biomedical applications or in the automotive industry, as well as in the manufacture of floating objects.

The study presented in [18] develops new possibilities for obtaining Mg matrix syntactic foams using porous natural volcanic rock with acceptable behavior both at room temperature and at high temperatures.

The performances of these materials can be improved by adjusting the structure and characteristics of volcanic rock particles. The results of the research presented in [19] confirm the possibility of obtaining syntactic foams from AZ61 magnesium alloy powders with a closed-cell structure by powder metallurgy processes and rapid microwave sintering using carbamide particles. The results confirm that lightweight foams are obtained with a buoyancy capacity.

The spontaneous motion of objects at a fluid interface, driven by local variations in surface tension, presents a compelling mechanism for autonomous, micro-scale locomotion. This self-actuation is governed by the Marangoni effect, where a gradient in surface tension generates shear stress on the fluid surface, propelling the object from regions of low surface tension to regions of high surface tension. Mathematically, this driving force is directly proportional to the surface tension gradient. Marangoni propulsion is highly attractive for applications such as micromixing, drug delivery, and environmental sensing due to its inherent efficiency and ability to convert chemical energy from a released agent into mechanical work [20].

The performance of a Marangoni swimmer—specifically its velocity and longevity—is critically dependent on the controlled release of the propelling agent (surfactant or solvent). Early swimmers often relied on solid, non-porous platforms, which typically resulted in a rapid initial burst of the agent followed by a sharp decay in performance as the agent reservoir was depleted or release became diffusion-limited [21]. Recent theoretical work has been instrumental in defining the hydrodynamic efficiency limits of this propulsion mechanism, emphasizing that efficient, sustained motion requires a continuous and uniform release profile [22].

To overcome these release limitations, the focus of materials research has shifted toward highly porous architectures that can act as both a structural body and a high-capacity, sustained reservoir [23]. Among these, metallic foams have emerged as a promising class of materials.

Despite this extensive work on their architecture and bulk properties, the direct application of metallic foams as active, self-propelled Marangoni swimmer bodies remains largely unexplored. While the porous structure of these foams is known to facilitate capillary action and hold significant volumes of fluid [24], their performance in generating a stable, sustained surface tension gradient for Marangoni-driven locomotion has not been systematically investigated.

In the proposed approach with magnesium foam swimmers, the entire porous surface could act as a distributed, self-regulating release valve driven by capillary action. The high porosity provides a large internal volume for fuel storage, enabling significantly longer operational lifetimes. Capillary forces within the fine pores continuously draw liquid from the reservoir to the surface, maintaining a stable surface tension gradient and steady velocity since the foam structure actively pulls and delivers the fuel to the contact surface using capillary pressure. Due to the very low pore size required for controlled ethanol release, the proposed foam will not be a bulk reservoir but a high-efficiency, passive distribution network. This solves the consistency and distribution problem but still introduces a drag penalty due to its inherent structural roughness. This structural roughness will be reduced by the manufacturing process, since the press and sinter fabrication method can result in samples with a high surface quality.

This work addresses that critical gap by leveraging the unique reservoir capacity and tailored release characteristics of metallic foams to engineer high-performance Marangoni swimmers.

## 2. Materials and Methods

### 2.1. Raw Materials

For the preparation of magnesium perlite syntactic foams, fine magnesium particles (<45 µm) and expanded perlite with a density of around 0.25 g/cm^3^ and a size range of 0.2–0.4 mm were utilized. Perlite is a natural, volcanic glass with a significant fraction of structurally bonded water molecules that expand this material when heated to high temperatures. If expanded completely, a very low density can be obtained [25]. After the EDS analysis, the oxide composition calculated from the corresponding metal atoms present is as follows: 75 wt.% SiO_2_, 14 wt.% Al_2_O_3_, 4 wt.% K_2_O, 3 wt.% Na_2_O, 1.3 wt.% CaO, 1 wt.% Fe_2_O_3_, and some minor traces of other oxides.

**Figure 1:**
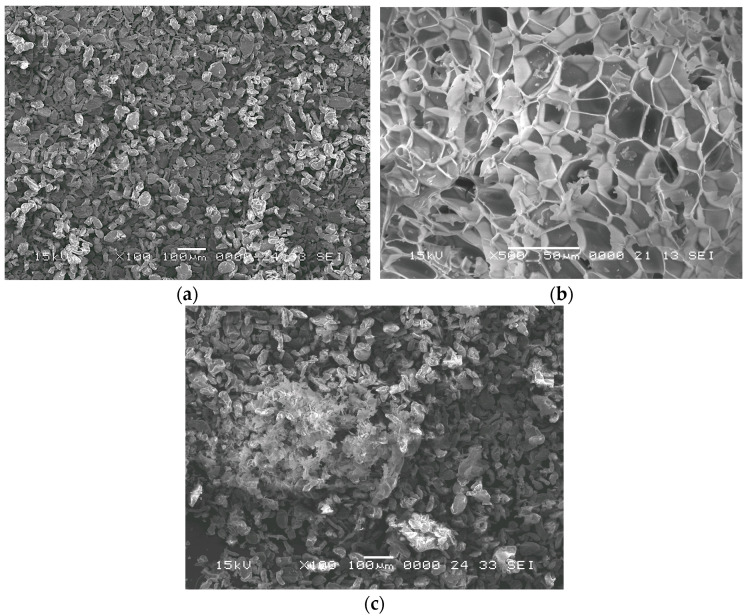
SEM images of the (**a**) magnesium powder and (**b**) expanded perlite particle, along with (**c**) the homogenized mixture.

Different ratios of magnesium particles and expanded perlite particles (60%, 70%, and 80% vol. of perlite particles) were produced. The starting mixtures presented a homogenous blend after 15 min of mixing in a home made spatial Turbula-type mixer, as shown by the SEM images in Figure 1. In Figure 1b, the image of a perlite particle in an expanded state is shown. The polyhedral cell pores resulting from the expansion of the perlite particles can be observed. No specific measures were taken regarding the atmosphere in which the magnesium powder and the perlite particles were homogenized.

### 2.2. Preparation of the Syntactic Foams

**Figure 2:**
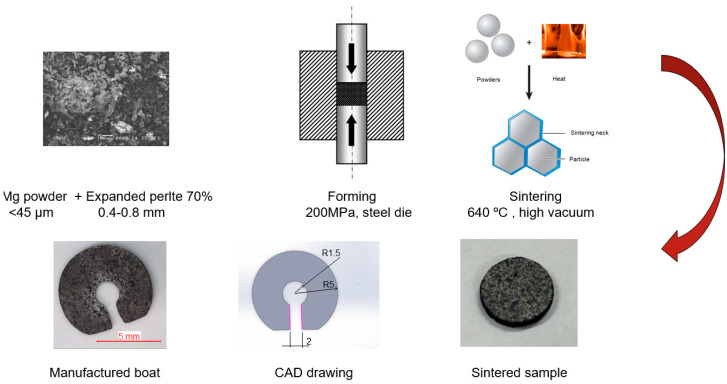
Schematic depiction of the manufacturing process of the Marangoni swimmer.

The appropriate quantities of magnesium powder and expanded perlite mix were cold-pressed in a hardened steel die with 200 or 300 MPa (Figure 2). The pressures used assured a good contact between the magnesium particles, although some densification of the perlite particles occurred. At lower pressures, the contact between the metallic particles was insufficient to produce decent sintering between the particles. All samples were cylindrical, with a diameter after sintering of 11.4 mm and approx. 1 mm in height.

Sintering of the particles was performed in a vacuum (~5 × 10^−6^ torr) at a temperature close to the melting point of the magnesium particles. The sintering temperature was set to 640 °C and was measured outside the vacuumed quartz tube holding the samples, giving a slightly underestimated temperature reading. The hold time was set to 30 min after some initial trials. All measurements were taken on three samples; their mean values are given in the manuscript. After the sintering process, good sintering necks were formed, although the sintering degree remained in the initial stage of sintering. Due to the magnesium’s high saturated vapor pressure, some weight loss was observed (<3%).

Most of the sintered samples (60% and 70%) had good mechanical properties, allowing their machining into the desired shape. The machining was conducted on a tabletop milling machine, where the central hole had a diameter of 3 mm and a channel with a width of 1.5 mm. As a propellant reservoir, a high-porosity polyurethane (PU) foam was used. The foam was cut into an ~5 mm side square and placed over the central hole, and a certain amount (3 mg) of propellant mixture was added.

### 2.3. Used Characterization Techniques

The density of the samples was determined by measuring their mass and calculating the cylindrical volume from dimensions taken with a caliper (0.1 mm precision). Porosity was subsequently estimated by comparing the measured density to the calculated mixture density, using Equation (1):

P = 1 − ρ_s_/ρ_m_ (1)

where P is the porosity, ρ_s_ is the sample’s density, and ρ_m_ is the magnesium-expanded perlite mixture’s theoretical density.

The morphology (shape) and local chemical composition of the particles were characterized using Scanning Electron Microscopy (SEM) coupled with Energy-Dispersive X-ray Spectroscopy (EDS) on a Jeol 5600 LV system-Akishima, Tokyo, Japan. Fresh fracture surfaces of the fired, uncoated samples were also analyzed using SEM-EDS. The EDS analysis utilized the ZAF correction standards implemented within the AZTEC 4.0 software. The pore size distribution was measured on a Thermo Scientific Pascal 140 mercury porosimeter (Thermo Electron, Waltham, MA, USA).

The contact angle was measured using optical tensiometry. This technique relies on analyzing the shape of a small water droplet placed on the sample surface, with the angle determined by analysis of the captured image.

**Figure 3:**
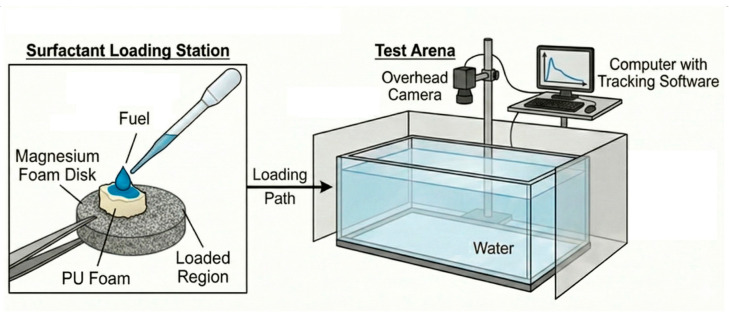
Schematic depiction of the movement test setup.

The microboat’s motion was recorded using a Canon PowerShot SX50 HS digital camera (Canon Inc., Tokyo, Japan) at a frame rate of 30 fps. The resulting videos were analyzed using publicly available image analysis software (ImageJ v. 1.54q, Kinovea 2023.1.2) to determine performance metrics. The velocity was measured using the Kinovea software. The video was taken from above the water tank, from which the distance covered was measured in pixels and was transformed into millimeters using an A3-sized graph paper reference (Figure 3). The time base was taken from the frame rate of the video. A simple model was applied to estimate the volumetric flow rate of ethanol as it was released from the aft (rear) of the swimmer [19]. The volumetric flow rate can be approximated by scaling the surface velocity by the cross-sectional area:

Q = v_max_ ∗ A (2)

where Q—volumetric flow rate, v_max_—maximum velocity, A—area of the slot (2 mm^2^).

## 3. Results and Discussion

### 3.1. Microstructural Characterization

In all cases, samples with high porosity were obtained. Since the aim of this paper was to make a Marangoni-type swimmer, its density must be below 1 g/cm^3^, and it must have good mechanical properties, which allows the manufacture of the desired shape.

Looking the data presented in Table 1, one can easily observe that the only viable candidate for the Marangoni surfer is the sample made of the 70% porosity mixture. The density decrease when using the 80% porosity mixture is significant; however, the limited numbers of sintering necks generated by the magnesium particles allow only reduced mechanical properties since the expanded perlite, although a ceramic, does not positively impact the mechanical properties. Increasing the magnesium faction to 40% improves workability. This improvement, however, increases the density to values that do not permit flotation.

**Figure 4:**
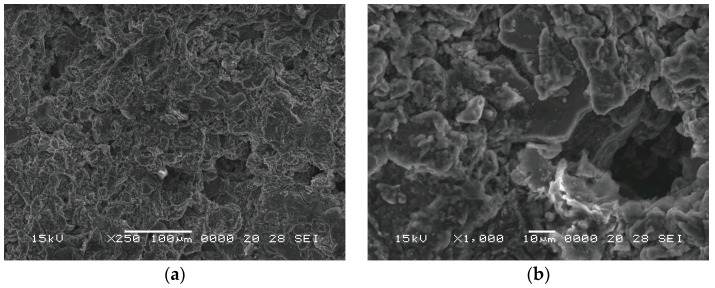
SEM images of the syntactic foam at different magnifications (**a**) ×250 and (**b**) ×1000.

The porosity is composed of “large” pores generated by the added perlite particles and small pores as voids between the magnesium particles, which are specific to powder metallurgy techniques. In Figure 4, images of the macroporous structure of syntactic magnesium foam are presented.

**Figure 5:**
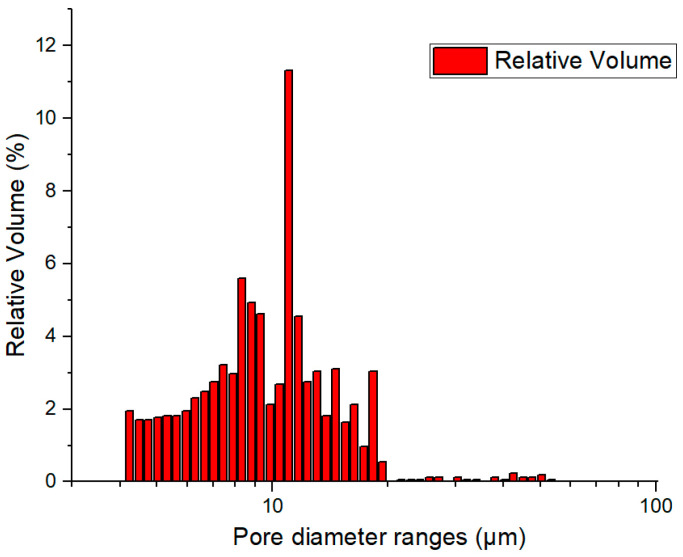
Typical pore size distribution of the manufactured foams.

The large pores (“large”) are the cellular pores resulting from the porous nature of perlite particles. These pores consistently reduce the samples’ density. The small pores are numerous but their influence on the final porosity is reduced, and they are formed between the particles of the powder mixture because of the sintering process. The size of the large pores is determined by the size of the porous cells of the perlite particles, while that of the small pores is determined by the size of the interparticle spacing of the magnesium particles. It is found from the SEM images that the pore size is less than 10 µm. The mercury porosimetry analysis is presented in Figure 5. As the SEM analysis predicted, in the pore size distribution, there are only small pores, all of them under 10 µm. This measurement incorporates all interconnected pores from between the magnesium particles but also from within the expanded perlite. With these small pores, water infiltration into the porous structure is prevented; furthermore, the porosity serves only to reduce the density, while it does not present a risk of sinking the swimmer during operation.

The specific macrostructure of the metal foam is ensured precisely by the presence of the expanded cellular pores of perlite. Foams with a density of 0.9 g/cm^3^ and a porosity of 48% were obtained.

### 3.2. Contact Angle Measurement

**Figure 6:**
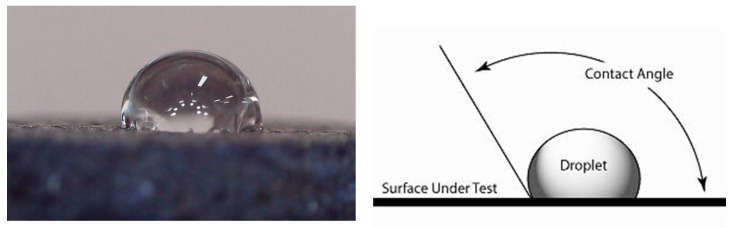
Contact angles of the water and the principle of its measurement.

The need to know the contact angle for a foam used as a Marangoni-type swimmer is motivated by its direct relevance to the interfacial tension gradients driving the motion. It can be used quantify the foam’s interaction with the liquid surface, which is the direct mechanism for the Marangoni propulsion. The manufactured foams have small enough pores to impede spontaneous impregnation with water. The surface characteristics of the foam are repellent enough for the foam to be considered water repellent and to have a high wetting angle of 120° (Figure 6). In the case of the alcohol, the behavior is inversed; it presents a low wetting angle of 23°. This difference suggests high driving for the Marangoni-type propulsion since for a simple swimmer, like a disk that releases a surfactant, the force is proportional to the surface tension difference:

(3) F~Δσ×L

where Δσ is the surface tension difference, and L is the swimmer’s size [26]

### 3.3. Marangoni-Induced Propulsion

This Marangoni swimmer design utilizes a composite structure that strategically separates the functions of bulk fuel storage and surface distribution by combining two different foam materials: polyurethane foam and magnesium foam.

The primary function of the PU foam is to serve as the bulk storage reservoir for the ethanol propellant. The PU foam’s high porosity and low density provide the swimmer with an extended operational lifetime. This PU foam is placed over the central hole, outside the water.

The magnesium foam—an open-cell structure with a carefully controlled pore connectivity and smaller pore size—is the structural support and passive distribution network. It draws ethanol from the PU core and spreads it uniformly across the contact perimeter using capillary forces. This continuous, regulated distribution ensures a constant supply of ethanol to the interface, which is crucial for maintaining a stable and uniform surface tension gradient for the swimmer.

**Figure 7:**
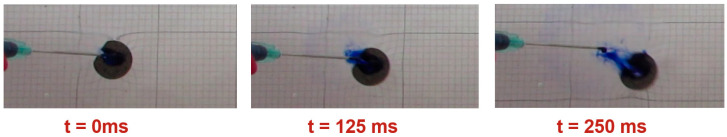
Recorded images presenting the first part of the swimmer’s movement.

The propulsion of the metallic foam surfer is achieved (Figure 7) through a dual-layer system: the polyurethane (PU) foam acts as the reservoir and primary diffuser of the ethanol, creating the necessary surface tension gradient at the liquid–air interface, while the metallic foam provides the structure, rigid support, and hydrodynamic profile. The observed velocity of approximately 37 mm/s is thus a result of the Marangoni thrust generated by the ethanol field, as it spreads from the PU reservoir through the manufactured channel outwards, and the significant viscous drag imposed by the rigid, porous metallic body. Since the metallic foam is the structure defining the physical boundary, its intrinsic roughness and low pore size will dictate the stability of the dynamic contact line as the entire unit moves. Instabilities in the contact line are responsible for the observed deviation from a linear movement.

The viscous drag results from the shear stress between the fluid and the wetted surface of the swimmer. It is caused by the viscosity of the water in the present case. It is proportional to the wetted surface area and the properties of the boundary layer. Since the metallic foam is not permeable to water, its inherent roughness will increase the skin friction drag compared to a smooth, non-porous body of the same shape. By increasing the pore size or the porosity, we are increasing the surface roughness and so the friction forces. A small reduction in the forces is achievable by increasing the porosity and diminishes the contact angle. For the typically small, slow-moving Marangoni swimmer, the flow is often laminar, and friction drag is the dominant component of the viscous forces.

This release of the surface tension-modifying liquid can be used also to release different agents in a controlled manner. When adding 5 or 10% of ink to the ethyl-alcohol, we evidenced its influence on the movement characteristics of the Marangoni surfer.

### 3.4. Movement Analysis

**Figure 8:**
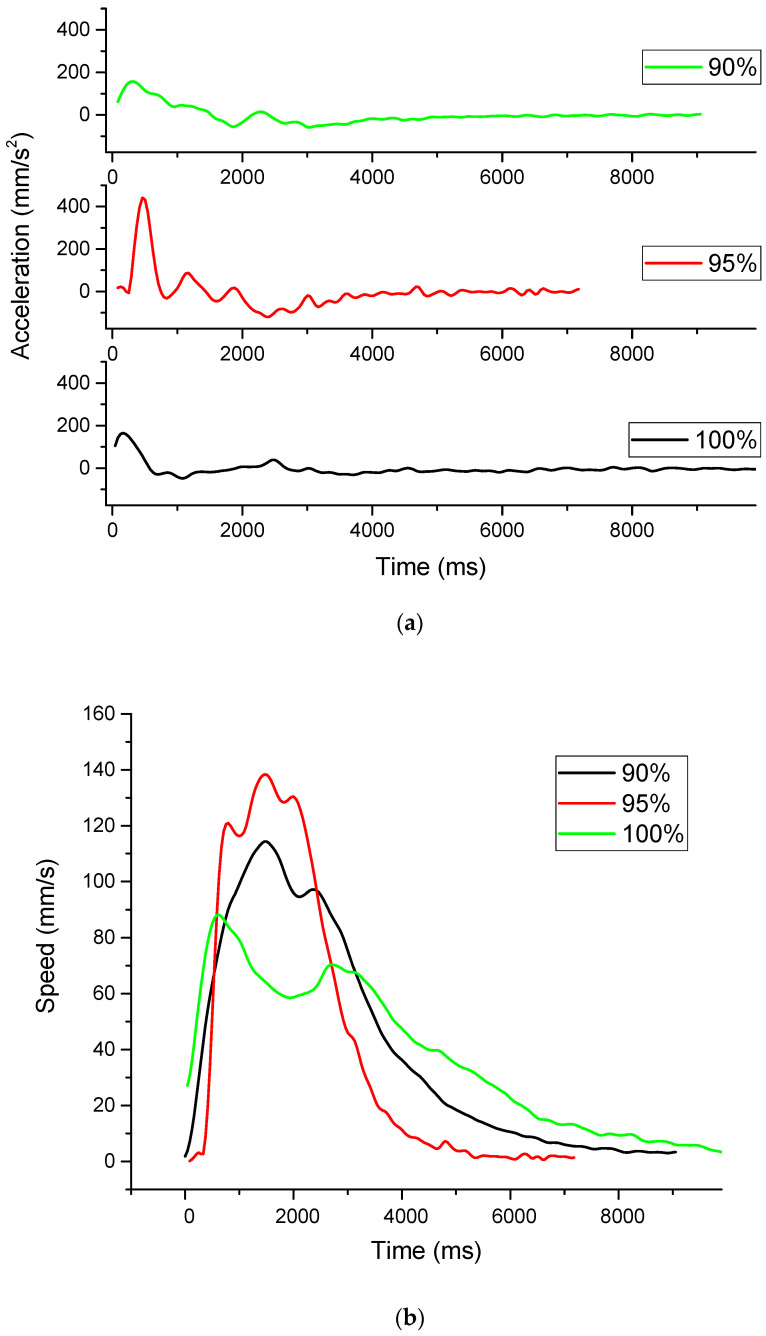

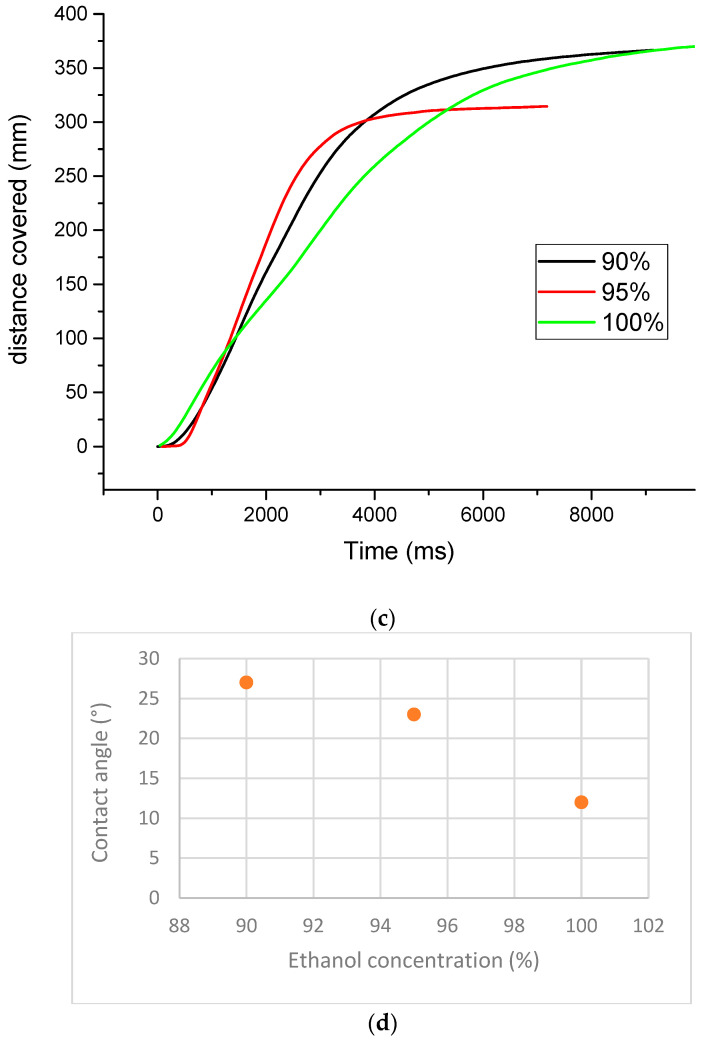
Movement characteristics of the Marangoni swimmer with time (**a**)—acceleration, (**b**)—speed, distance covered (**c**) and the wetting angle as a function of ethanol concentration—(**d**).

The movement analysis clearly demonstrates that ethanol dilution within the polyurethane reservoir is the primary controlling factor for the Marangoni propulsive force, thereby governing the swimmer’s traveled distance, velocity, and kinetic stability (Figure 8). The inconsistent release of ethanol is clearly visible in the graphs presenting the evolution of speed and the calculated acceleration. The highest acceleration will give the highest top speed; however, more force is consumed than in the case of slower acceleration. This is evident in the case of the samples with the ethanol 95%–ink 5% samples, where the distance covered is significantly shorter.

### 3.5. Estimation of the Flow Rate

The analysis of the flow rate is crucial for understanding both the hydrodynamic drag imposed by the metallic foam and the mass transfer kinetics of the surfactant from the polyurethane reservoir. This rate can be estimated by considering the fluid flow induced by the Marangoni stresses and the rate of ethanol consumption.

The volumetric flow rate of the surrounding liquid driven by the Marangoni stresses can be approximated by scaling the surface velocity by a characteristic cross-sectional area giving an estimate for fluid transport Equation (2).

This approximation proposed by A.D. Akinwekomi et al. [19] is a simple and convenient way for determining the volumetric flow rate, where Q—volumetric flow rate v_max_—maximum velocity, A—area of the slot (2 mm^2^) from which ethanol diffused into water. The results are presented in Figure 8.

To maximize the efficiency of the propulsion, all the ethanol must be diverted around the metallic foam. Although the pores of the metallic foam are small, the low surface tension of the ethanol allows for a portion of it to penetrate the highly porous structure, slightly increasing the overall drag profile and lowering the Marangoni thrust.

The depletion of ethanol (Q_ethanol_) from the PU reservoir gives the most direct estimation of the required surfactant release flow rate. This rate can be calculated from the known initial concentration, the reservoir size, and the time required for the movement to stop or drop to a negligible velocity.

(4) $$Q_{ethanol}=\frac{Mass\;of\;ethanol\;consumed}{Time\;of\;movement}$$

The calculated Q_ethanol_ should be proportional to the V_max_. A higher release rate leads to a stronger, more sustained Δσ and thus higher speeds. Any deviation from a simple proportionality indicates that the system has reached a saturation point where the hydrodynamic drag of the metallic foam becomes the dominant limiting factor.

**Figure 9:**
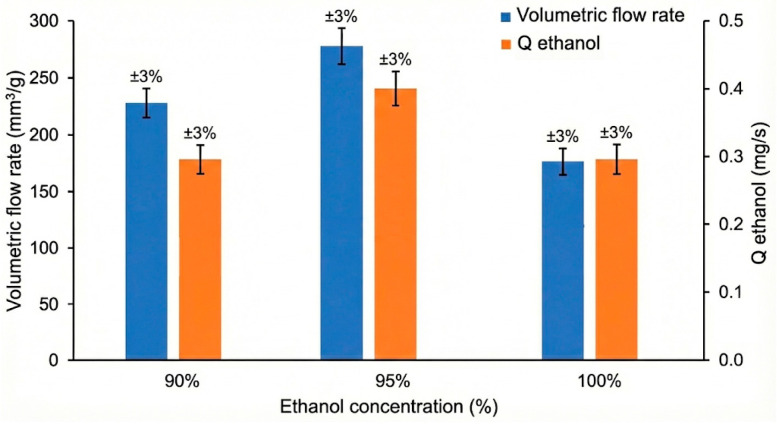
Comparison of the ethanol flow rates vs. concentration.

In Figure 9, some key information is visible. The peak performance should be at a 95% concentration: both the mass flow rate and the volumetric flow rate achieve their maximum values at the 95% ethanol concentration. The volumetric flow rate peaks at 277.2 mm^3^/s and Q_ethanol_ peaks at 0.396 mg/s.

Despite the Q_ethanol_ values being similar for the 90% and 100% concentrations, the volumetric flow rate is significantly lower at 100% (176.4 mm^3^) than at 90% (227.8 mm^3^). This suggests that factors other than just the mass release rate may be limiting the overall fluid flow rate at the highest concentration.

The 95% ethanol (95% ethanol + 5% ink) mixture achieving the highest acceleration and speed, rather than the 100% or 90% mixtures, is likely due to an ideal balance of surface tension reduction and release stabilization achieved by the 5% ink concentration. The propulsive force of a Marangoni swimmer is directly proportional to the steepness of the surface tension difference created as the fuel spreads away from the swimmer. Plain ink is not just a dye; it contains surfactants and other active compounds that dramatically lower the surface tension of the liquid, even in small amounts. These ink components act as co-surfactants alongside ethanol and control its release from the PU foam’s pores [27,28].

In the case of the 95% ethanol concentration mixture, there is an optimal amount of these surfactants to boost the surface tension reduction capability of ethanol without causing detrimental effects. However, the release of the ethanol mixture from the reservoir’s pores is discontinuous; before the next quantity of propellant is released, the previously released droplet was already spent and the swimmer is in a deceleration stage, losing momentum. The next droplet must reverse this negative acceleration, and so, although it has a higher acceleration capacity, the distance covered was shorter than in the other two cases. In those cases, the release of the propellant is closer to a continuous release pattern, and since the effects of the propellant droplets are overlapping, the deceleration stages are reduced. The higher surface tension difference in the case of pure ethanol means the swimmer moves for a longer time, and so, although having a lower top speed, it travels further. Further investigations are needed to optimize the pore size distribution of the reservoir to maximize the Marangoni swimmers’ performance.

## 4. Conclusions

By pressing and sintering the mixture of magnesium powder and expanded perlite, low-density syntactic metal foams were obtained, which can be used in various applications, such as the manufacture of swimmers for mass transfer in liquids using Marangoni propulsion.

By modifying the ethanol concentration, a variation in the surface tension was evidenced by measuring the wetting angle on the swimmer. The ethanol mixtures had low wetting angles as opposed to the water, together with the floatability of the foam, creating the premises for Marangoni-type propulsion.

The observed mean velocities were 37, 44, and 40 mm/s (for the 100%, 95%, and 90% concentrations) generated by the Marangoni thrust of the ethanol field. The top speeds measured during the movement analysis were considerably higher, at 88, 138, and 114 mm/s (same concentrations as above). The high speeds of the last two concentrations were maintained only for approximately 3.5 s, and after that, the speed dropped sharply. In contrast, the 100% ethanol had the lowest high speed; however, it was able to maintain it for a longer duration (almost twice as long as the other concentrations). This longer duration is most likely due to a lower surface tension, which permits a high Marangoni thrust, combined with an almost continuous and slower decay rate.

The main contribution of the presented results is a simple and reliable way of manufacturing Marangoni swimmers, which can reach high speeds using common surface tension modifications. As a further step in the manufacturing, we suggest shape optimization and, of course, the optimization of the pores for the usage of the whole porosity as a fuel reservoir.

## Figures and Tables

**Table 1:** Sample densities and workability.

Target Porosity	Applied Pressure	Density	Porosity	Workability
60%	200 MPa	1.07–1.15 g/cm^3^	34–38%	Good
70%	200 MPa	0.9–0.92 g/cm^3^	47–48%	Good
80%	200 MPa	0.65–0.7 g/cm^3^	58–60%	Inacceptable
80%	300 MPa	0.7–0.73 g/cm^3^	60–63%	Inacceptable

## Data Availability

The original contributions presented in this study are included in the article, and further inquiries can be directed to the corresponding author.
